# Therapeutic potential and mechanistic insights of silibinin targeting cancer-associated fibroblasts in colorectal cancer

**DOI:** 10.3389/fphar.2025.1527871

**Published:** 2025-04-02

**Authors:** Shenglan He, Jianmei Ji, Kaisi Zhu, Youlan Chen, Xiaowen Xu

**Affiliations:** ^1^ Department of Gastroenterology, Shuguang Hospital Affiliated to Shanghai University of Traditional Chinese Medicine, Shanghai, China; ^2^ Department of Digestive Endoscopy, Shuguang Hospital Affiliated to Shanghai University of Traditional Chinese Medicine, Shanghai, China; ^3^ Department of Anesthesiology, Changhai Hospital, Navy Medical University, Shanghai, China; ^4^ Institute of Integrated Traditional Chinese and Western Medicine Digestive Diseases, Shuguang Hospital, Shanghai University of Traditional Chinese Medicine, Shanghai, China

**Keywords:** silibinin, colorectal cancer, cancer-associated fibroblasts, anti-proliferation, antimigration

## Abstract

**Objective:**

This study aims to elucidate the role of SB in inhibiting CRC progression by targeting CAFs and elucidating the underlying mechanisms.

**Methods:**

In this study, a spontaneous CRC model induced by AOM/DSS was used to evaluate the effects of SB on CAFs. Mice were treated with SB, and tumor burden was assessed by colon length. CAFs were isolated post-treatment for transcriptomic analysis to identify differentially expressed genes, with molecular docking providing *in silico* evidence of SB’s binding to target proteins. CAFs changes were further examined through HE staining, IHC, and assays for cell viability, colony formation, and migration. Integrated bioinformatic analysis elucidated the mechanistic role of SB in CAFs-mediated CRC progression.

**Results:**

*In vivo* studies showed that SB effectively reduced POSTN and α-SMA protein levels in CAFs in AOM/DSS-induced CRC mice. Consistently, *in vitro* experiments demonstrated that SB significantly decreased both protein and mRNA levels of α-SMA and POSTN in fibroblasts (colonic myofibroblast CCD-18Co cell lines.) co-cultured with CRC cell lines (human colorectal adenocarcinoma SW480 and RKO cell lines). SB also inhibited proliferation, colony formation, and migration of CCD-18Co cells. Transcriptomic and integrated bioinformatic analyses further suggested that SB exerts therapeutic effects on CAFs in CRC by modulating key target pathways.

**Conclusion:**

These results demonstrated that SB holds promise as a therapeutic agent for targeting CAFs in CRC. This study advances our understanding of SB’s mechanisms, particularly its inhibitory effects on CAFs proliferation, colony formation, and migration.

## 1 Introduction

Colorectal cancer (CRC) is among the most frequently diagnosed cancers globally and stands as the second most common cause of cancer-related mortality (Sung et al., 2021). To develop effective therapies for CRC, it is vital to gain a comprehensive understanding of the fundamental pathophysiological mechanisms that drive its progression. Recent evidence has increasingly emphasized the tumor microenvironment (TME) as a critical factor in the oncogenesis and progression of cancer (Xiao and Yu, 2021). In particular, intercellular communication within the TME has emerged as a key regulator influencing the malignant behavior of CRC cells (Chen et al., 2015).

Therefore, to advance effective therapies for CRC, a comprehensive understanding of the fundamental pathophysiological mechanisms is essential, driving its progression is essential. In recent years, growing evidence has highlighted TME as the critical factor in oncogenesis and progression of cancer (Xiao and Yu, 2021). Specifically, intercellular communication within the TME has emerged as a key regulator of the malignant behavior of CRC cells (Chen et al., 2015). Within the CRC TME, cancer-associated fibroblasts (CAFs) are particularly prominent among the various stromal components. Numerous studies underscore the substantial role of CAFs in CRC progression; however, the mechanisms by which CAFs drive CRC progression remain inadequately understood, highlighting the need for further investigation.

Natural products, known for their multiple molecular targets and favorable safety profiles, are emerging as valuable sources of novel therapeutic agents (Yao et al., 2021). The rising adoption of complementary and alternative medicine among cancer patients has generated increasing interest in botanical therapies, which are widely utilized as medicinal agents and dietary supplements. In China, a significant number of Chinese patent medicines and injections about anti-cancer have been developed from natural plants, represented by Cinobufacini injection, Compound Kushen injection, and Shen Fuzheng injection (Xu et al., 2024; Chen et al., 2003; Wu et al., 2024). These natural product-derived medications have been extensively used in clinical settings. Notably, the clinical effectiveness of Cinobufacini injection has been rigorously evaluated through randomized controlled trials, yielding robust evidence that supports its use in evidence-based medicine (Chen et al., 2003), demonstrating a substantial number of applications and notable therapeutic efficacy. Moreover, Silibinin (SB), a drug derived from natural sources, has attracted substantial interest for its promising therapeutic properties. Its pharmacological profile and natural origin position it as a valuable candidate for further research and potential clinical applications.

SB serves as the primary active ingredient in silymarin, an extract standardized from the seeds of *Silybum marianum*, commonly known as milk thistle. SB was approved by the China Food and Drug Administration in 2008 for hepatoprotective clinical applications. Available dosage forms include dietary supplements (capsules, tablets, and softgel) as well as an injectable formulation, highlighting SB’s hepatoprotective effects, favorable safety profile, diverse therapeutic applications, and minimal side effects (Raina et al., 2016; Hoh et al., 2006). Owing to its therapeutic effects, SB’s established well-documented safety record and widespread use as a dietary supplement have made it a promising candidate for further investigation in CRC treatment research. Thus, SB shows significant potential as a therapeutic agent in CRC treatment due to its direct anti-tumor effects, ability to enhance the efficacy of other treatments, and potential to improve patient outcomes by prolonging survival and increasing disease control rates.

Extensive research on SB has investigated its diverse range of effects. SB has gained attention for its diverse anti-tumor effects including promoting apoptosis and autophagy, while inhibiting angiogenesis and cell migration (Tuli et al., 2021). Despite its potential significance, research on SB’s regulatory effects on the TME remains limited, and its mechanisms of action, particularly in relation to CAFs, are poorly understood. CAFs are a critical component of the TME and play a key role in driving CRC progression through their interactions with tumor cells and other stromal components. This knowledge gap highlights the need for further investigation into how SB influences the TME, including its impact on CAF-mediated processes. Therefore, this study aims to elucidate the molecular pathways through which SB inhibits CRC progression, with a focus on its regulatory effects on CAFs, and to establish a scientific foundation for its therapeutic potential.

## 2 Methods and materials

### 2.1 Spontaneous CRC model establishment and treatment grouping

Male C57BL/6 mice (6–8 weeks), received intraperitoneal injections of azoxymethane (AOM) (Supplementary Table S1 reagents). Fourteen mice were randomly divided into Model or SB group. Both groups were initially provided with regular drinking water for a 7-day acclimation phase. Following this, the mice were given 2% dextran sulfate sodium (DSS) in their drinking water for 7 days, succeeded by a return to regular water for 14 days. This cycle was repeated three times. On day 15, the SB group started treatment with SB given by oral gavage at 500 mg/kg daily, 5 days per week, while the Model group received normal saline. This regimen continued until the completion of the third cycle, covering a total period of 10 weeks. On day 70, mice were sacrificed, and colon tissues were collected for further analysis. Measurements included colon length, tumor count, tumor size, and the isolation of colon CAFs. All procedures involving animals received approval from the Laboratory Animal Ethics Committee (Approval No. PZSHUTCM2302080008) and were performed in compliance with License No. SCXK(Hu)2020-0009.

### 2.2 Fibroblast isolation and validation

100 mg of Type I collagenase was dissolved in 1 mL of 0.1 mol/L sterile PBS to make a 1.0% (W/V) stock solution, filtered through a 0.22 μm membrane for sterility. Tissue specimens were rinsed three times with sterile PBS containing antibiotics. Excess fat was removed, and then the tissue was cut into 1 mm^3^ pieces, incubated with collagenase I at 37°C in 5% CO_2_, and mixed every 0.5 h until digested. After that, Digestion was stopped by centrifugation (1,000 r/min, 5 min), followed by PBS washing. The pellet was resuspended in DMEM with 20% FBS and incubated in sterile glass bottles. After 72 h, the medium was replaced. Finally, Cells were cultured to 80%–90% confluence, digested with 0.25% trypsin, resuspended in DMEM with 10% FBS, and transferred for continued culture.

### 2.3 Western blotting

Protein concentrations were quantified using the Bradford assay. A 10 μg sample was applied to a 10% SDS-PAGE gel for electrophoretic separation, with subsequent transferred onto a polyvinylidene fluoride (PVDF) membrane. The upper layer contained concentrated glue maintained at a constant pressure of 80 V, while the lower layer contained separated glue held at a constant pressure of 120 V. The membrane was subjected to a constant current of 300 A for a duration of approximately 20 min. This setup continued until bromophenol blue reached the bottom of the gel. After that, membrane was incubated overnight at 4°C with primary antibodies such as Glyceraldehyde-3-phosphate dehydrogenase (GAPDH), Alpha-smooth muscle actin (α-SMA), and Periostin (POSTN) (Supplementary Table S2) after being blocked with 5% milk. Following primary antibody incubation, the membrane was exposed to a secondary antibody for 1 h at room temperature, with three subsequent washes in TBS-T buffer, each lasting 10 min. Detection of target proteins was achieved using a chemiluminescent system.

### 2.4 Hematoxylin and eosin (HE) and Masson staining

For HE and Masson staining in tissue, colorectal tissue and tumor samples were fixed in 4% formaldehyde for 48 h, followed by dehydration, clearing, paraffin embedding, and sectioning into 4-μm thick slices. The tissue sections were then dewaxed, rehydrated, and stained using hematoxylin and eosin or Masson’s trichrome stain (Supplementary Table S3. INSTRUMENTS and SOFTWARE). The stained slides were subsequently imaged and analyzed under a light microscope.

For HE staining in cells, fibroblasts were seeded into Petri dishes and fixed with 4% paraformaldehyde for 15 min. Subsequently, samples were washed 3 times in phosphate-buffered saline, with each wash lasting for 3 min. Hematoxylin staining solution was then applied to the samples at room temperature for 4 min, with three subsequent rinses in double-distilled water. Eosin staining solution was subsequently applied for 3 min, followed by another rinse. Finally, the samples were mounted for microscopic examination using a CX43 microscope.

### 2.5 Immunohistochemistry (IHC)

In the IHC experiment, fibroblasts were fixed onto glass slides using 4% paraformaldehyde. Moreover, the slides were incubated with 5% bovine serum albumin for 30 min and incubated overnight at 4°C with the primary antibody α-SMA. The following day, secondary antibody IgG was applied for 30 min, followed by DAB for chromogenic detection. Furthermore, the slides were stained with hematoxylin to highlight the nuclei for visualization. Under the microscope, the target protein appeared brown, while the nuclei were stained purple.

### 2.6 RNA extraction and RNA-Sequencing

Total RNA was isolated with TRIzol reagent (Rio et al., 2010). Genes showing a fold change of ≥2.0 with an unadjusted *p*-value of ≤0.05 were considered significantly upregulated or downregulated. Each time point in the microarray analysis included three biological replicates. Differentially expressed genes (DEGs) were analyzed between Model and SB group, including volcano plots of DEGs, heatmap of the top 30 genes, protein-protein interaction (PPI), gene ontology (GO), and Kyoto Encyclopedia of Genes and Genomes (KEGG) pathway.

### 2.7 Analyze RNA-Seq

The single-cell RNA sequencing (scRNA-seq) data of 11 adjacent normal tissues and 15 tumor tissues from CRC patients were retrieved from the GSE231559 dataset, which is accessible through the Gene Expression Omnibus (GEO) database at the following URL: https://www.ncbi.nlm.nih.gov/geo/query/acc.cgi?acc=GSE231559. The R software package Seurat (5.0.1) was used to analyze scRNA-seq data. In short, nFeature_RNA >200 and nFeature_RNA <2,500 and percent. mt < 15 was set to filter the cell. First, we used UMAP for cell clustering. Then, the HumanPrimaryCellAtlasData-based R software package singleR (2.4.1) was used for cell annotation, followed by the CellMarker database for manual annotation of different clusters. Employing the “limma-voom' software package for the analysis of RNA-sequencing data, the criteria for selecting differentially expressed genes included a minimum fold change of two and a maximum p-value of 0.05. This approach ensures a stringent selection process that highlights genes with significant expression changes. Finally, cellchat (1.6.1) was used for the analysis of cell-cell communication.

### 2.8 Molecular docking

PECAM1, COL1A2, THBS2, COL3A1, POSTN, and Vimentin were key targets about fibroblast activation, and the molecular structure was obtained from PBD database (PBD:5YJG, 1GK4, 2KY5, 6FZV, 1YO8, and 5CTD) respectively. Molecular docking and binding energy assessments were performed using Home for Researchers (He et al., 2025) (https://www.home-for-researchers.com/index.html/).

### 2.9 Cell culture and co-culture system

The cell lines were sourced from Shanghai Leiyi Medical Technology, including human colorectal adenocarcinoma SW480 and RKO cell lines and colonic myofibroblast CCD-18Co cell lines. SW480 cells were grown in Dulbecco’s modified Eagle’s medium enriched with 10% fetal bovine serum (FBS) and 1% Penicillin-Streptomycin at 37°C with 5% CO_2_, while RKO cells were cultured in RPMI 1640 medium. CRC cells and CCD-18Co cells (3 × 10^4^ cells per well) were plated cells in upper and lower chamber of Transwell inserts for 7 days.

### 2.10 Cell viability, cytotoxicity and formation assay

Cell Counting Kit-8 was utilized to assess cell viability and Cytotoxicity. CCD-18Co (3,000 per well) in 100 μL of culture medium were planted in 96-well plates, followed by adhered for 12 h. In Cytotoxicity, Cells were seeded in 96-well plates at a density of 5 × 10^4^ cells per well and treated with increasing concentrations of SB (0, 6.25 μM, 12.5 μM, 25 μM, 50 μM, 100 μM, 200 μM, 400 μM) for 72h, and the concentrations of result was 46.67 μM which was used in followed experiments. Moreover, each well added 10 μL of CCK-8 solution and incubated for an additional 3 h. The microplate reader was configured to measure absorbance at 490 nm. In colony formation assay, CCD-18Co (3,000 cells per well) were seeded in 6-well plates, incubated for 10 days. Finally, the colonies were analyzed using ImageJ software.

### 2.11 Transwell migration assay

The migration capability of CCD-18Co cells was evaluated using Transwells in 24-well plates. The upper chamber added 100 µL suspension (1× 10^4^ cells), while the lower chamber added 600 µL of medium with 10% FBS, incubated for 24 h. Subsequently, the lower surface was fixed with 2% paraformaldehyde and stained with crystal violet. Images were captured under an inverted microscope from three random fields per well, and integrated optical density was analyzed using ImageJ software.

### 2.12 Quantitative real-time PCR

First-strand cDNA was synthesized from 1 μg of total RNA using the PrimeScript RT reagent kit. Real-time PCR analysis was then conducted with SYBR Premix Ex Taq™ on a 7,500 Fast Real-Time PCR detection system. Target gene mRNA expression levels were normalized to β-actin and calculated using the 2^−ΔΔCT^ method. Primer sequences are provided in Supplementary Table S4.

### 2.13 Database

We investigated the correlation between CAFs and expression of COL1A2, PTN and THBS2 and was obtained using the TIMER 2.0 database (http://timer.cistrome.org). Furthermore, we evaluated the survival impact of COL1A2, PTN and THBS2 in CRC using the Kaplan-Meier Plotter (http://kmplot.com) database.

### 2.14 Statistical analysis

Data analysis was conducted using Prism nine software. Results are presented as mean values ±SEM. Statistical differences between groups were evaluated using Student’s t-test, with *p*-values less than 0.05 considered statistically significant.

## 3 Results

### 3.1 Effect of SB on fibroblasts *in vivo*

**FIGURE 1:**
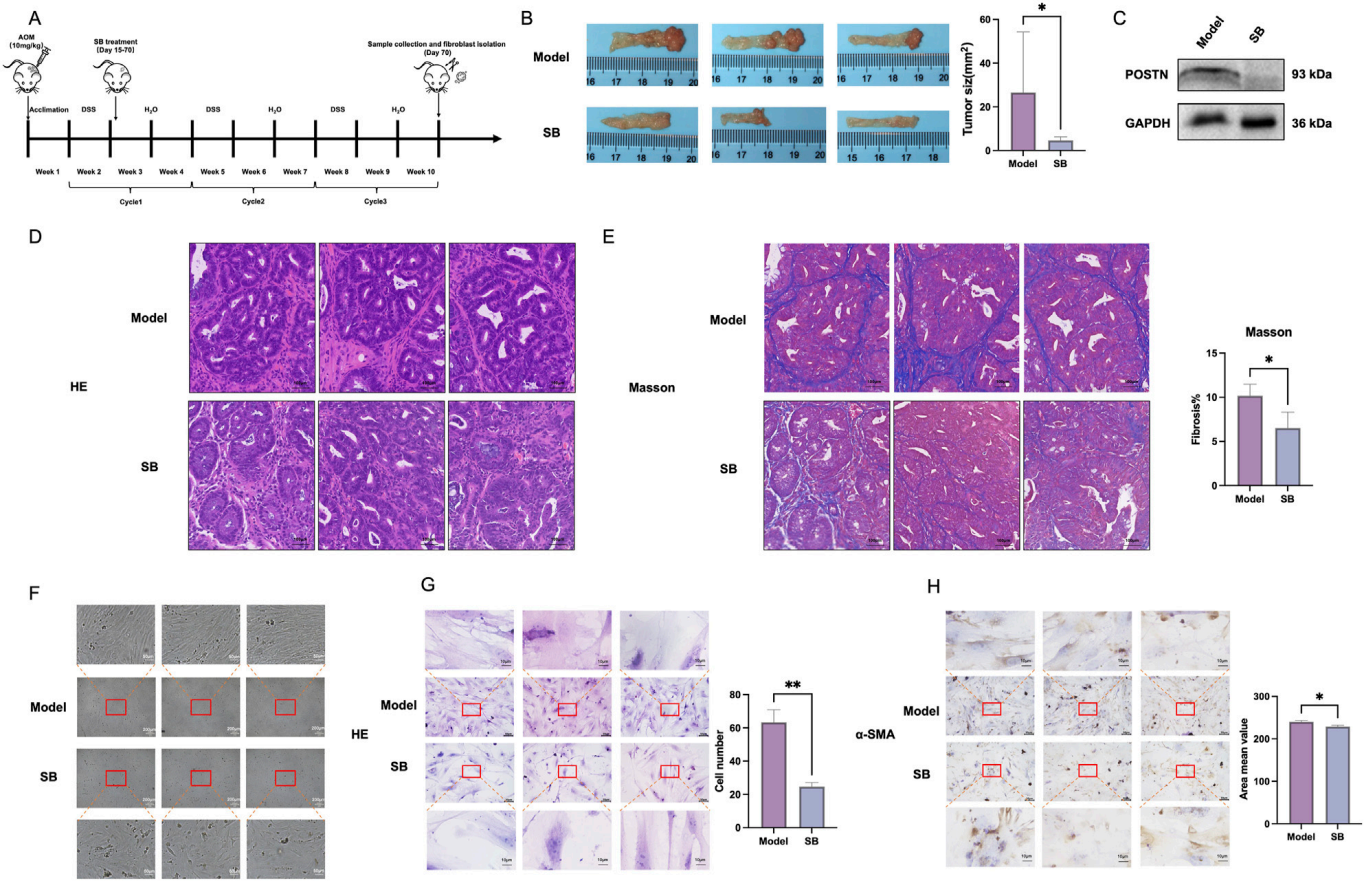
Effect of SB on fibroblasts *in vivo*. **(A)** Model establishment schematic diagram. **(B)** Length of tumors. **(C)** Western blotting. **(D)** HE staining (100 μm). **(E)** Masson staining (100 μm). **(F)** Morphology of fibroblasts (200 μm). **(G)** Representative photographs stained by HE (50 μm and 10 μm). **(H)** Representative photographs stained mIHC (50 μm and 10 μm). SB: Silibinin; HE, Hematoxylin-eosin; mIHC, Multiplex immunohistochemistry; *, *p* < 0.05, **, *p* < 0.01.

To assess the impact of SB on CRC, a murine model of spontaneous, chemically-induced CRC was created using DSS/AOM (Figure 1A). The findings showed that during the 55-day SB treatment period, the mice receiving SB had notably smaller tumors compared to those in the model group (*p* < 0.05, Figure 1B). Furthermore, SB also could reduce the expression of protein levels of POSTN (Figure 1C). Histological analysis using H&E staining demonstrated SB group exhibited more regular intestinal mucosal cells and crypt structures compared to model group (Figure 1D). Masson staining showed reduced fibrosis and improved tissue integrity in the SB group (Figure 1E). Morphology and HE staining showed the SB decreased the proliferation of fibroblast compared to model group (Figures 1F, G). In addition, IHC staining further revealed that expression of α-SMA was significantly reduced in SB treatment (Figure 1H).

### 3.2 Differential gene expression and pathway analysis of fibroblasts via transcriptomics

**FIGURE 2:**
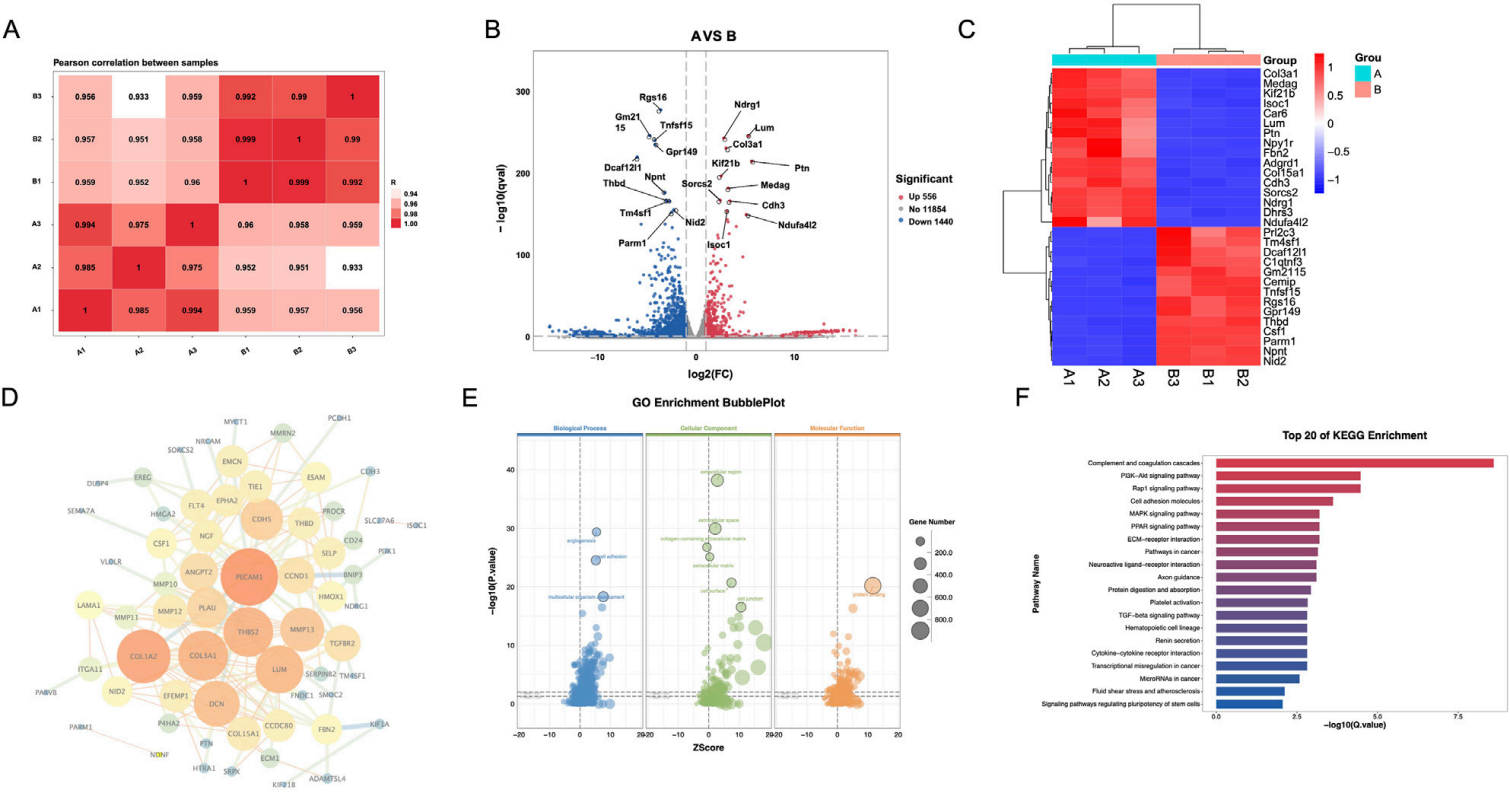
Differential gene expression and pathway analysis of CAFs via transcriptomics. **(A)** Pearson correlation between samples. **(B)** Volcano map. **(C)** Heatmap. **(D)** PPI network. **(E)** GO enrichment analysis. **(F)** KEGG enrichment analysis. A: Model; B: SB (Silibinin).

To evaluate differential genes in CRC fibroblasts between treatment groups, we first analyzed the pearson correlation between samples, which all of correlation between samples are above 0.933 (Figure 2A). In the volcano, the top five upregulated genes included genes Rgs16, Gm2115, Tnfsf15, Gpr149, and Dcaf12L1, and the top five downregulated gene Ndrg1, Lum, Col3a1, Kif21b, and Ptn (Figure 2B). A heatmap was generated to cluster the top 30 genes in each group, selected based on their p-values (Figure 2C), showed significant enrichment and strong association with Csf1, Col3a1, Tm4sf1, Rgs16, and C1qtnf3. Analysis of the PPI network identified the top five targets as PECAM1, COL1A2, THBS2, COL3A1, and LUM (Figure 2D). GO enrichment pathway revealed that SB is main associated with angiogenesis, extracellular, and protein blinding (Figure 2E). KEGG enrichment analysis indicated that the PI3K-AKT signaling pathway was the primary pathway enriched (Figure 2F).

### 3.3 Molecular docking of SB and CAFs maker targets

**FIGURE 3:**
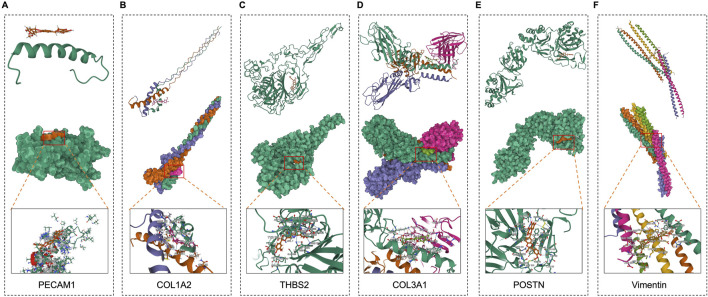
Molecular docking of SB and CAFs maker targets. **(A-F)** Molecular docking of between SB and targets, including PECAM1, COL1A2, THBS2, COL3A1, POSTN, and Vimentin.

To further evaluate the binding capacities of PPI network targets including PECAM1, COL1A2, THBS2, COL3A1 and important targets for fibrosis including POSTN and Vimentin (Figures 3A-D), we performed *in silico* analyses (Figures 3E,F). We demonstrated that SB for these targets had blinding enery of −84.343, −7.151, −9.443, −9.229, −8.426, and −7.778 kcal/mol, and blinding molecular are 2KY5, ARG690, GLU683, SER687, and LYS691(PECAM1); ALA48(COL1A2); THR682, ARG1111, TYR684, ASP945, ASP694, LYS1166, and HIS714(THBS2); ANS36, TYR67, and ASP58(COL3A1); CYS 44 and LYS 65(POSTN); AGR 381, SER 339, and THR 336(Vimentin). These results supported those targets are the potential targets of anti-CRC in SB.

### 3.4 Effect of SB on fibroblasts in co-culture

**FIGURE 4:**
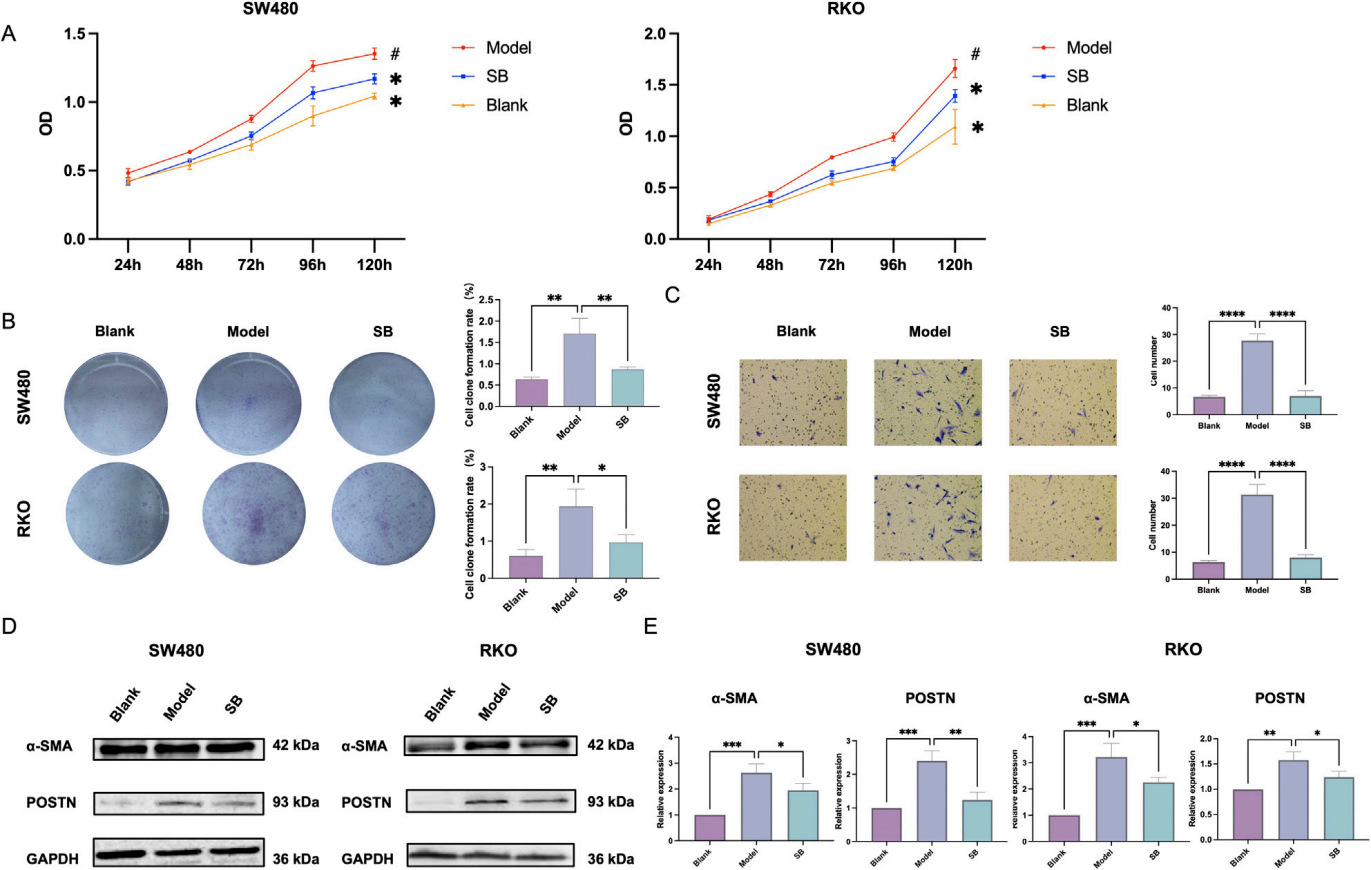
Effect of SB on fibroblasts in co-culture. **(A)** Proliferation in each group. **(B)** Cloning formation capabilities. **(C)**Transfection. **(D)** Western blotting. **(E)** mRNA expression in each group. SB: Silibinin; **, *p* < 0.01, ***, *p* < 0.001.

The co-culturing was divided into blank group, model group and SB group. To investigate the effects of SB on the biological functions of human colorectal fibroblasts, CCD-18Co fibroblasts were co-cultured with SW480 and RKO cells. Notably, the results indicated that SB decreased the proliferation, cloning formation, and migration of CCD-18Co cells in co-culture compared to model group (Figures 4A–C). Furthermore, SB was also able to decrease both mRNA and protein expression levels of α-SMA and POSTN in fibroblasts (Figures 4D, E). In summary, our observations indicate that SB treatment significantly decreases the expression of fibrosis-associated markers.

### 3.5 Analysis of SB’s anti-cancer function via fibroblasts in TME

**FIGURE 5:**
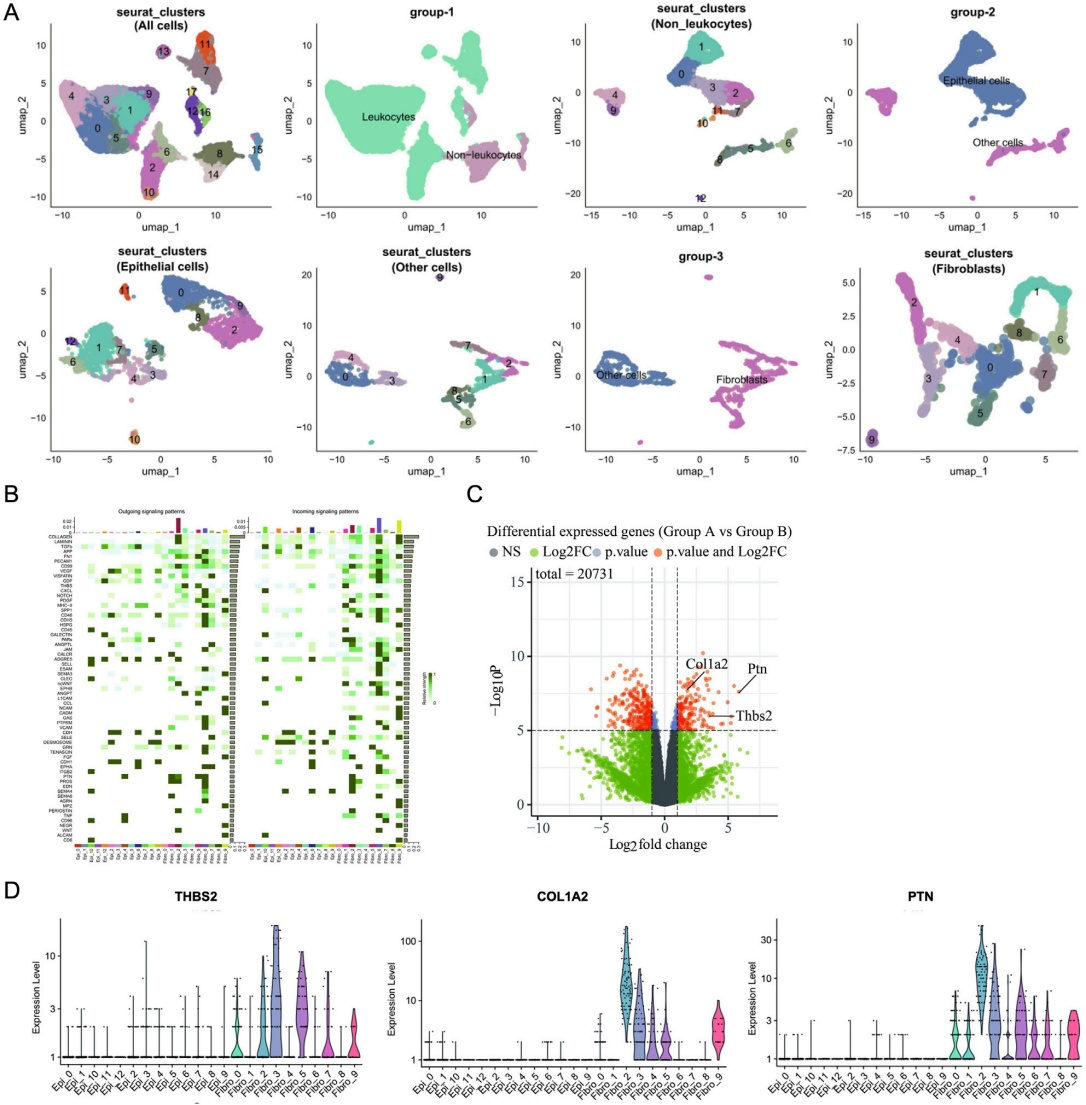
Analysis of SB’s anti-cancer function via fibroblasts in TME. **(A)** Cellular Subtyping and Clustering Analysis Using Seurat. **(B)** Heatmap. **(C)** Volcano map. **(D)** Differential Signal Expression in Fibroblast Subclusters. A: Model; B: SB (Silibinin).

Analysis of RNA-seq and scRNA-seq data further presents cellular subtyping and clustering analysis using seurat, including UMAP clustering of all cells, leukocyte and non-leukocyte separation, non-leukocytes, epithelial and non-epithelial cell, epithelial cells, non-epithelial cells, fibroblast and non-fibroblast cell, fibroblasts (Figure 5A). Utilizing the R package ‘CellChat’ to calculate the output signals and input signals of cellular communication between different epithelial cell subpopulations and distinct fibroblast subpopulations (Figure 5B). RNA-seq was used to compare the expression profiles of fibroblasts in group model and SB. We found that SB may regulate cell communication between fibroblasts and epithelial cells through COL1A2, PTN and THBS2 (Figure 5C). In addition, differential signal expression in fibroblast subclusters were compared. THBS2 was high expression in fibroblast subclusters 0, 2, 3, 5, 7, and 9. COL1A2 was high expression in fibroblast subclusters 0, 2, 3, 4, 5, and 9. PTN was high expression in fibroblast subclusters 0, 1, 2, 3, 5, 6, 7, and 9 (Figure 5D).

### 3.6 Expression and prognosis analysis in the database

**FIGURE 6:**
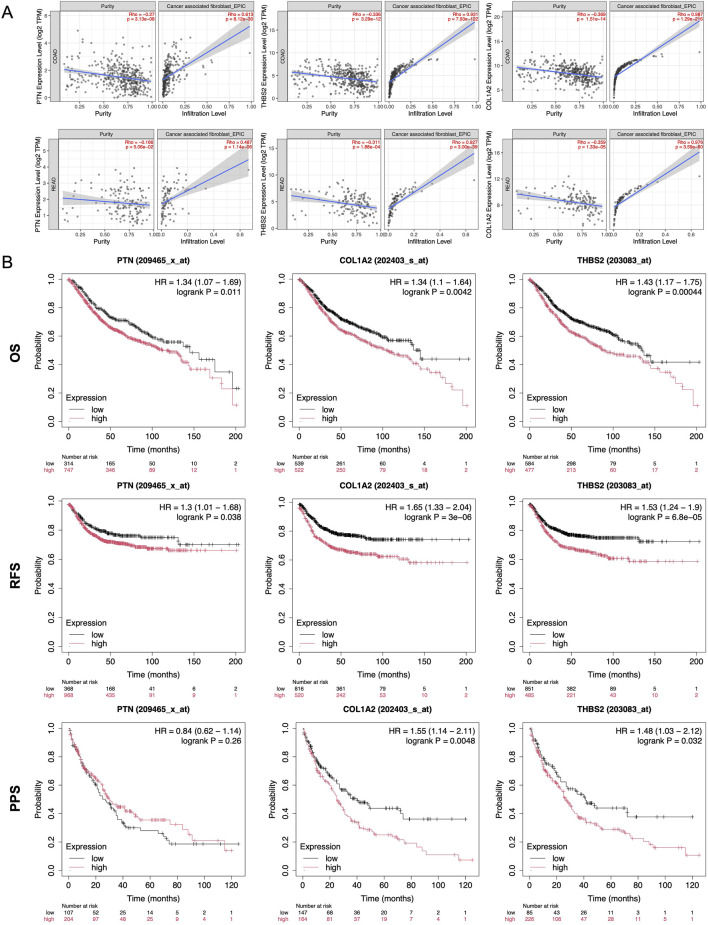
Expression and prognosis analysis in the database. **(A)** Infiltration Ptn, Thba2, and Col1a2 of from TIMER2.0 database in CAFs. **(B)** Kaplan-Meier survival curve analysis of Ptn, Thba2, and Col1a2 in overall survival, recurrence-free survival, and post progression survival of CRC. **, *p* < 0.01, ***, *p* < 0.001.

The TIMER 2.0 database shows a positive correlation between PTN, THBS2, and COL1A2 expression and immune cell infiltration in CAFs of colorectal adenocarcinoma (COAD) and rectal adenocarcinoma (READ) (Figure 6A). For those targets, EPIC Rho of COAD are 0.613, 0.931, and 0.987, and EPIC Rho of READ are 0.487, 0.927, and 0.976, respectively. These targets are closely related to CAFs in TME of CRC (*p* < 0.05).

Kaplan-Meier plotter curve revealed that patients expressing higher levels of PTN, THBS2, and COL1A2 exhibited shorter survival compared to those with lower levels in overall survival, recurrence-free survival, and post progression survival (Figure 6B). These findings underscore its potential as both a prognostic biomarker and a therapeutic target in the management of CRC.

## 4 Discussion

SB has garnered considerable attention and has been extensively investigated in various biological and preclinical studies for its efficacy against cancer with minimal side effects (Yao et al., 2021; Tuli et al., 2021). SB is widely recognized for its hepatoprotective effects against drug- and alcohol-induced liver damage (Yao et al., 2021). Studies have shown that SB also exhibits significant anti-tumor properties, acting on tumor cells through mechanisms such as induction of apoptosis, regulation of autophagy, and modulation of microRNAs. These pharmacological actions have been extensively documented in CRC models in number of studies (Jahanafrooz et al., 2018). Additionally, SB demonstrates anti-tumor efficacy across multiple stages of tumor progression within the TME, influencing key processes including angiogenesis, inflammatory responses, immune cell modulation, extracellular matrix remodeling, and the activity of tumor-associated fibroblasts. This broad therapeutic profile has made SB a focal point of interest in CRC research (He et al., 2025).

TME in CRC includes cellular and noncellular ingredient that surround and interact with tumor cells, undergoing dynamic changes throughout each stage of tumor formation and progression. These changes contribute to critical processes, including tumor growth, angiogenesis, and epithelial-mesenchymal transition (Joyce, 2005; Chen et al., 2015). Non-tumor components within the TME, which display greater genetic stability than tumor cells, play essential roles in the regulation of drug resistance (Boumahdi and Sauvage, 2019). Studies suggest that modulating the TME can mitigate acquired drug resistance, inhibit tumor progression, and ultimately improve patient survival (Xiao and Yu, 2021). Among these components, CAFs are particularly significant, as they contribute to both drug resistance and tumor growth (Sahai et al., 2020). Consequently, targeting CAFs represents a promising therapeutic strategy for enhancing treatment outcomes in CRC.

In addition, studies have shown that monocyte chemotactic protein-1 (MCP-1) is a critical factor in the cancer cell microenvironment that enhances the invasiveness of prostate cancer cells. Notably, treatment with SB significantly decreased MCP-1 expression in CAFs by suppressing the DNA-binding activity of transcriptional regulators AP-1 and NF-κB, which control MCP-1 expression (Ting et al., 2016). Interestingly, SB impaired the interaction between PCA cells and fibronectin, thereby inhibiting cell motility, invasiveness, and survival (Ting et al., 2016). Although there are limited studies on the effects of SB on CAFs within the TME, its role in modulating these cells remains largely unexplored, indicating a need for further research. SB has demonstrated various therapeutic effects in CRC, including inhibition of tumor cell proliferation, anti-inflammatory action, suppression of angiogenesis, induction of apoptosis, and chemopreventive properties.

Extensive research has explored the distribution and metabolic pathways of SB in drug delivery systems. Pharmacokinetic studies highlight promising drug formulations that improve SB’s absorption and bioavailability (Pérez-Sánchez, et al., 2019; Flaig et al., 2007). Findings indicate that SB is primarily distributed to the liver and intestines, supporting its therapeutic applications in hepatic and gastrointestinal conditions (Zhao and Agarwal, 1999). Additionally, substantial evidence underscores SB’s potential in targeting CRC tumors, reinforcing its viability for localized cancer therapy (Singh et al., 2008; Sameri et al., 2021).

It is worth noting that, in current research, most animal models involve the use of immunodeficient nude mice, which limits the comprehensive evaluation of the TME. Although APC-mutant models have been employed to provide insights into CRC development, these models may still be inadequate for assessing the complete progression of CRC and the role of an inflammatory TME throughout the disease course. *In vivo* experiments, one study shows an AOM-induced colon cancer model in A/J mice was used to evaluate the effects of SB, indicating that SB significantly reduced VEGF and iNOS expression in tumor tissues and demonstrating its anti-angiogenic properties (Ravichandran et al., 2010). Additionally, an APC^min/+^ murine model, which develops spontaneous intestinal adenomas, was employed to investigate the chemopreventive impact of SB on CRC, and showed that SB selectively inhibited the formation of nestin-positive microvessels within polyps, effectively reducing polyp formation in the model mice (Rajamanickam, et al., 2010). In the present study, we observed that SB treatment reduced tumor numbers in a DSS/AOM-induced CRC murine model which could better simulate TME. Additionally, HE and IHC analyses showed decreased fibroblast activation, underscoring SB’s therapeutic potential in CRC.

Our RNA-Seq analysis identified Platelet endothelial cell adhesion molecule-1 (PECAM1), type I collagen α2 chain (COL1A2), THBS2 (thrombospondin-2), and type III collagen α1 chain (COL3A1) as potential therapeutic targets in CRC, with each gene being closely associated with fibroblast activity. PECAM1 is predominantly expressed in endothelial cells (Mamdouh et al., 2003). Although PECAM1 is not a specific marker for fibroblasts, it may indirectly interact with CAFs by influencing endothelial cells involved in angiogenesis and inflammatory processes within the TME (Sahai et al., 2020). COL1A2 and COL3A1 are essential collagen proteins synthesized and secreted by fibroblasts, which play a critical role in maintaining the extracellular matrix (Szabo et al., 2023). Through collagen production, fibroblasts contribute to tissue structure and stability, with COL1A2 expression serving as an indicator of fibroblast activity and functional status. In conjunction with COL1A2, COL3A1 supports tissue elasticity and resilience by forming a complex collagen network. CAFs, through the synthesis of collagens, contribute to the formation of a dense tumor stroma. This stromal network not only provides structural support and protection for tumor cells but also creates a physical barrier that may limit the penetration and effectiveness of anti-cancer drugs (Szabo et al., 2023). THBS2 is another fibroblast-associated protein that regulates fibroblast activity by binding to cell surface receptors. This protein plays a role in various physiological and pathological processes, including wound healing, tissue repair, and fibrosis (Yang et al., 2022). Given these associations, SB may exert its anti-CRC effects by modulating CAFs activity, thereby influencing the composition and behavior of the TME.

This study investigated the potential of SB to inhibit fibroblast in CRC through bioinformatics analyses and *in vivo* experiments. Our results demonstrate that SB significantly reduces the expression of key CAFs markers, including α-SMA and POSTN, at both protein and mRNA levels, indicating its potential to suppress CAFs activity as a therapeutic strategy for CRC. Further bioinformatics analyses were performed by using CRC dataset to explore SB treatment in CAFs, and identified THBS2, COL1A2, and PTN as molecular targets of SB in CRC treatment. These targets appear to play pivotal roles as “sender,” “mediator,” and “receiver” within the TME, positioning THBS2, COL1A2, and PTN as primary regulators of CAFs activity in response to SB treatment. Importantly, these targets not only directly affect CAFs but also interact with other cell types within the TME, forming a complex network of mutual regulation. For instance, THBS protein family, particularly THBS2, is strongly associated with CAFs, and elevated THBS2 expression is linked to poorer patient prognosis (Yang et al., 2022). THBS2 can influence tumor cell proliferation, migration, and invasion, contributing to CRC progression (Liu et al., 2024). Additionally, PTN, a growth factor, promotes extracellular matrix secretion and cytokine production, which collectively support TME formation by enhancing CAFs proliferation and migration (Chen et al., 2024). All together, these findings highlight THBS2, COL1A2, and PTN as promising therapeutic targets for CRC. Detailed exploration of these targets may clarify the mechanisms underlying SB’s anti-tumor effects and contribute to the development of innovative diagnostic and therapeutic approaches for CRC and potentially other cancers.

This study has several limitations. First, our focus was primarily on CAFs, and we did not conduct a comprehensive evaluation of the entire TME, leaving other components for potential future investigation. Second, our analysis centered on a specific subset of fibroblasts, with other subtypes remaining unexplored. Additionally, in animal studies, we exclusively examined the oral route of administration, without assessing alternative delivery methods. Lastly, the limited quantity of sorted primary cells restricted the extent of downstream analyses. In this study, RNA-Seq analysis was employed to identify and validate potential targets and signaling pathways through which SB may exert therapeutic effects in CRC. Our findings suggest that SB regulates CAFs activation by downregulating key CAFs biomarkers, including α-SMA and POSTN, at both the protein and mRNA levels, thereby impacting CAFs growth and proliferation. SB emerges as a promising therapeutic candidate for CRC treatment by specifically targeting THBS, COL1A2, and PTN—key regulators involved in CAFs activation. These results highlight SB’s potential for modulating fibroblast activity and disrupting CAFs-mediated processes within the TME, paving the way for novel strategies in CRC therapy.

## 5 Conclusion

The present study results provide novel insights of SB for CRC treatment and shed light on potential strategies for modulating CAFs pathogenesis in the TME. SB emerges as a promising therapeutic candidate for inhibiting CAFs activation by downregulating key CAFs biomarkers α-SMA and POSTN, thus offering potential avenues for modulating fibroblast activation towards CAFs in CRC therapy.

## Data Availability

The datasets presented in this study can be found in online repositories. The names of the repository/repositories and accession number(s) can be found below: https://www.ncbi.nlm.nih.gov/geo/, GSE231559.

## Abbreviations

AOM, Azoxymethane; α-SMA, Alpha-smooth muscle actin; CAFs, Cancer-associated fibroblasts; CCK8, Cell counting kit-8; COL1A2, type I collagen α2 chain; COL3A1, type III collagen α1 chain; CRC, Colorectal cancer; DEGs, Differentially expressed genes; DMEM, Dulbecco’s modified Eagle’s medium; DSS, Dextran sodium sulfate; GAPDH, Glyceraldehyde-3-phosphate dehydrogenase; GO, Gene Ontology; HE, Hematoxylin-eosin; KEGG, Kyoto Gene and Genome Encyclopedia; MCP-1, monocyte chemotactic protein-1; mIHC, Multiplex immunohistochemistry; PECAM1, Platelet endothelial cell adhesion molecule-1; POSTN, Periostin; PPI, Protein-Protein Interaction; PTN, Pleiotrophin; RPMI, Roswell Park Memorial Institute; THBS2, thrombospondin-2; TME, Tumor microenvironment.
