# Supplementary figures and images for: Therapeutic potential and mechanistic insights of silibinin targeting cancer-associated fibroblasts in colorectal cancer
Source: Front Pharmacol. 2025 Apr 2;16:1527871. doi: 10.3389/fphar.2025.1527871 (PMC12000085; doi:10.3389/fphar.2025.1527871)

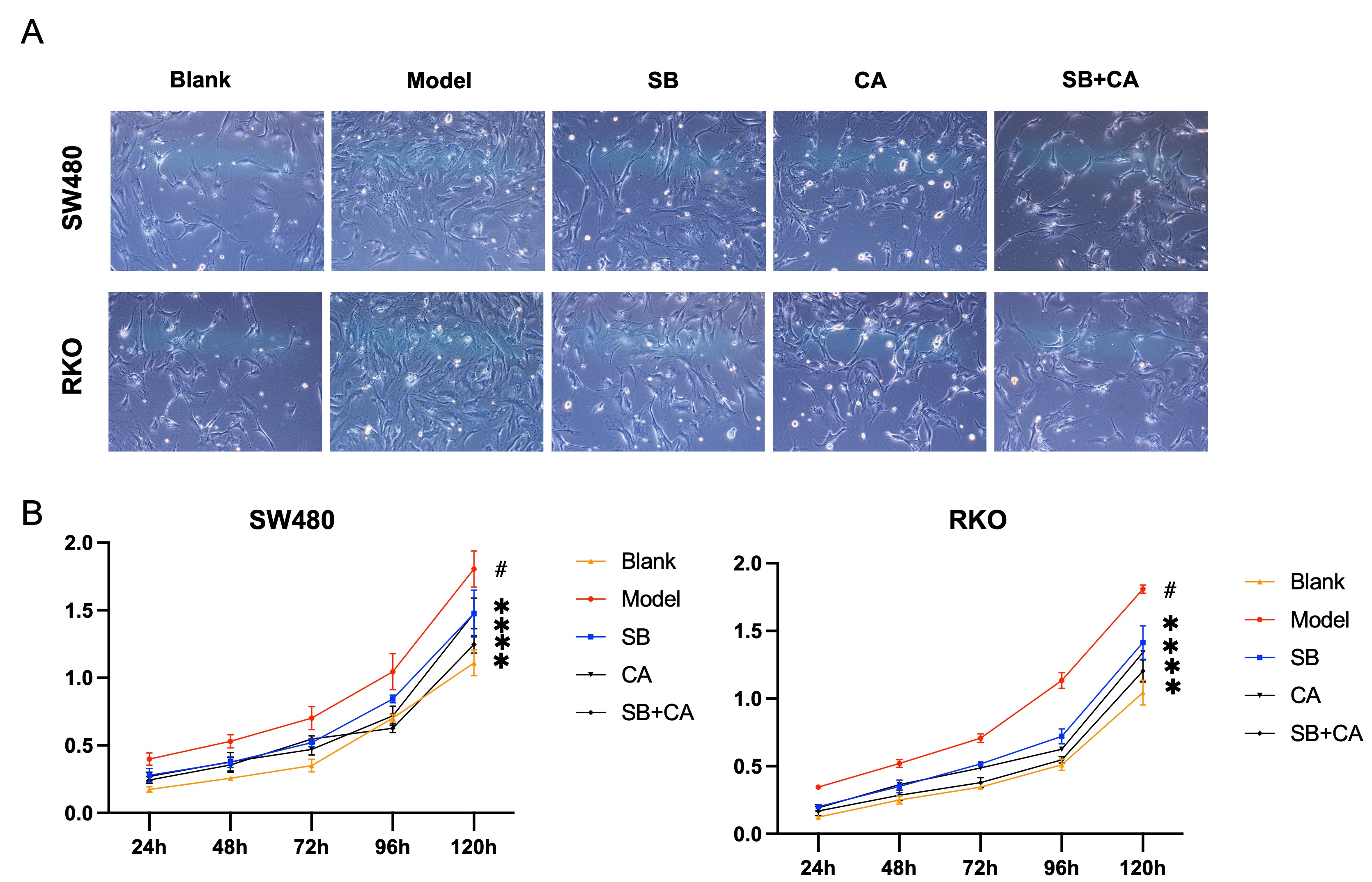

Supplement: Supplementary file 1 [file Image1.jpeg]
